# The role of lncRNAs in AKI and CKD: Molecular mechanisms, biomarkers, and potential therapeutic targets

**DOI:** 10.1016/j.gendis.2024.101509

**Published:** 2024-12-30

**Authors:** Minhui Zheng, Zixuan Yang, Lei Shi, Liyuan Zhao, Kelan Liu, Naping Tang

**Affiliations:** a Shanghai Innostar Bio-Technology Co., Ltd., China State Institute of Pharmaceutical Industry, Shanghai 201203, China; b Anhui University of Traditional Chinese Medicine, Hefei, Anhui 230000, China; c Yangtze Delta Drug Advanced Research Institute, Yangtze Delta Pharmaceutical College, Nantong, Jiangsu 226133, China; d Intensive Care Unit, Liyang People's Hospital, Liyang, Jiangsu 213300, China

**Keywords:** Acute kidney injury, Biomarker, Chronic kidney disease, Exosome, lncRNA, Renal fibrosis

## Abstract

Exosomes, a type of extracellular vesicle, are commonly found in different body fluids and are rich in nucleic acids (circRNA, lncRNAs, miRNAs, mRNAs, tRNAs, *etc*.), proteins, and lipids. They are involved in intercellular communication. lncRNAs are responsible for the modulation of gene expression, thus affecting the pathological process of kidney injury. This review summarizes the latest knowledge on the roles of exosome lncRNAs and circulating lncRNAs in the pathogenesis, biomarker discovery, and treatment of chronic kidney disease, renal fibrosis, and acute kidney injury, providing an overview of novel regulatory approaches and lncRNA delivery systems.

## Introduction

Exosomes are nano-sized membrane vesicles that carry lipids, proteins, nucleic acids, and other bioactive substances, which have been first identified in sheep reticulocytes.^1^ Exosomes originate as small luminal vesicles within multivesicular bodies (MVBs) and subsequently fuse with the cytoplasmic membrane to expel these nano-sized vesicles into the extracellular space. Previously, it was considered as “waste” for cellular elimination, but recent research has found that it can participate in intercellular communication. Exosomes are released from different cells and body fluids, including platelets, immune cells, smooth muscle cells, endothelial cells, *etc*. When they are released from host cells into recipient cells, exosomes can mediate the biological activities of the recipient cells through the lipids, proteins, and nucleic acids they carry. Three pathways are mainly involved in exosome-regulated intercellular communication, including receptor–ligand interaction, endocytosis, and membrane fusion.^2^

lncRNA is a form of non-coding RNA with >200 nucleotides in length. Some lncRNAs have no or weak protein-coding ability and are less conserved when compared with mRNAs.^3^ Most of the lncRNAs have spatiotemporal gene expression during tissue development and differentiation, for example, 1300 lncRNAs have been studied for mice and they showed differential expression patterns in different parts of brain tissues.^4^ Growing evidence indicates that lncRNAs play a crucial role in regulating gene expression, by which their transcription leads to gene activation or silencing.

Approximately 13 million new acute kidney injury (AKI) cases were reported globally each year, accounting for 10%–15% and 50% of patients admitted to hospitals and intensive care units, respectively.^5^^,^^6^ AKI is characterized by a sudden decline in kidney function, a decrease in glomerular filtration rate, and increases in blood urea nitrogen and serum creatinine, though elevated serum creatinine level is not a sensitive indicator of kidney damage. Given that AKI primarily damages the nephron's tubules, glomerular metrics such as glomerular filtration rate and their proxies like serum creatinine level, can only suggest a tubule injury indirectly.^7^ Hence, serum creatine level is markedly increased after a reduction of glomerular filtration rate or progression of tubular injury. If not detected or treated early, AKI will continue to progress to chronic renal inflammation and tubulointerstitial fibrosis, eventually resulting in chronic kidney disease (CKD). Therefore, it is of utmost urgency to discover sensitive, specific, and stable biomarkers. This review summarizes the latest knowledge of the roles of exosomal lncRNAs and circulating lncRNAs in the pathogenesis, biomarker discovery, and therapeutic strategies of different kidney diseases, especially AKI, renal fibrosis, and CKD. Finally, we will also outline novel regulatory approaches for targeting exosomal lncRNAs and lncRNA delivery systems.

## Biogenesis of exosomes

**Figure 1:**
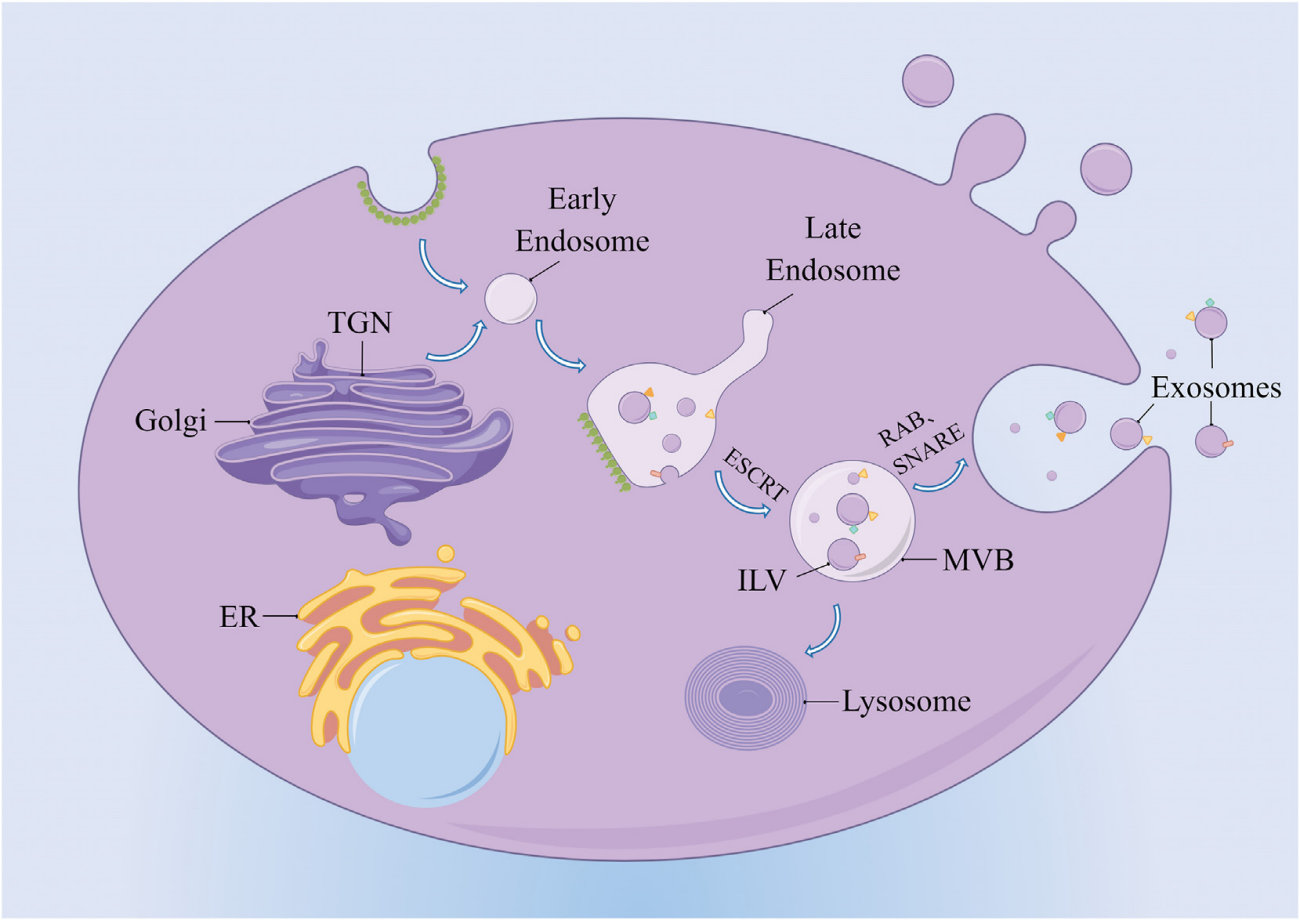
Biogenesis of the exosomes (by Figdraw.com). Early endosomes are formed by the inward budding of the plasma membrane, or in some cases, from the trans-Golgi network. Exosome biogenesis is regulated by ESCRT-dependent and ESCRT-independent pathways. After multivesicular bodies containing multiple intraluminal vesicles are formed, they are directed to the plasma membrane via a Rab GTPase-dependent multistep process where they undergo an SNARE-mediated plasma membrane fusion event followed by secretion into the extracellular space. In addition, they can fuse with the lysosome or apoptotic bodies. TGN, trans-Golgi network; ER, endoplasmic reticulum; MVE, multivesicular endosome.

The endosomal pathway is involved in the progression of diseases (Fig. 1). Early endosomes are formed through an invagination of the cell membrane. The vesicular organelles derived from the trans-Golgi network budding can fuse with early endosomes to form late endosomes, resulting in the invagination of the endosomal membrane into the lumen and the formation of intraluminal vesicles (ILVs). When the late endosomes contain multiple ILVs, they become MVBs.^8^

The production of ILVs and MVBs requires the assistance of the endosomal sorting complex required for transport (ESCRT), a protein complex that is located on the cytoplasmic side of the endosome. It is mainly involved in sorting specific components into ILVs, which can form the precursors of exosomes.^9^ The four core complexes (ESCRT-0, –I, –II, and –III) that recognize ubiquitinated membrane proteins and trigger membrane budding, which results in the creation of MVBs and ILVs, are the primary players in this process. ESCRT-0 detects and classifies ubiquitinated endosomal membrane proteins, to put it briefly. Additional ESCRT-I and ESCRT–II binding induces the endosomal membrane to bud inward, thus enabling the capture of various cargoes. Subsequently, the ESCRT-III complex can undergo deubiquitination and cleave the budding vesicles.^10^ Early endosomes are generated by these budding vesicles, which later mature into MVBs and late endosomes. Besides, in the absence of ESCRT and with the assistance of ceramides, lipids, heat shock proteins, or tetraspanins, the cells can create ILVs and MVBs.^11^ Neutral sphingomyelinase (n SMase) hydrolyzes sphingolipids to produce ceramide, and inhibition of this enzyme decreases the production of ceramides. Inhibition of this enzyme can attenuate ceramide production, which in turn prevents the inward budding of MVB membranes and reduces exosome production.^12^ This suggests that ceramides can be produced independently of ESCRT.

After intracellular production of MVBs, degradative MVB fuses with lysosomes, leading to its degradation and fusion with the cytoplasmic membrane as well as the secretion of exosomes to the extracellular compartment. The release of exosomes into the extracellular compartment relies mainly on the auxiliary roles of the RAB GTPases and soluble NSF-attachment protein receptor (SNARE). RAB GTPases are molecular switches that modulate the transport of intracellular vesicles. The SNARE proteins are responsible for the fusion of MVBs with the plasma membrane, leading to the secretion of exosomes.^13^ VAMP7, a SNARE protein that fuses secretory lysosomes with the plasma membrane, can induce the production of extracellular vehicles in K562 leukemia cells.^14^

## Contents and functions of exosomes

To date, approximately 600 lipids, 40,000 nucleic acids, and 350,000 proteins are detected in different exosomes. Proteins are the most crucial components of exosomes, which are considered to be of cytoplasmic, membrane fusion, and endocytic origin. Exosomes originating from the endolysosomal compartment are more likely to be enriched in major histocompatibility complex (MHC) class II and tetraspanins CD-37, -53, −63, −81, and −82.^15^ Several accessory proteins, such as programmed cell death 6 interacting protein (Alix) and tumor susceptibility gene 101 protein (Tsg101), are also involved in the function of the ESCRT pathway. These proteins can be directed by MVBs to lysosomes for decomposition. The exosomes are consisted of ESCRT proteins, TSG101, Alix, and chaperones (*e.g.*, Hcs70 and Hsp90), irrespective of their cellular origin.^16^

## The isolation methods of exosomes

Different approaches have been proposed for exosomal isolation, including size-exclusion chromatography, ultrafiltration, immunoaffinity, density-gradient ultracentrifugation, differential centrifugation, and precipitation-based methods.

## Differential centrifugation

Differential centrifugation is the most common technology at present, and its principle is based on the differences in size and density between exosomes and impurities in the samples. The centrifugation speed is typically set at 300 *g* for cell extraction at 4 °C for 10 min and then 2000 *g* for extracting cell debris at 4 °C for 10 min. After that, the speed is increased to 10,000 *g*, to separate protein pellets and larger extracellular vesicles as precipitations according to their density and size. Lastly, the supernatants are exposed to 100,000 *g* for 1–3 h for exosomal separation.^17^ This approach has several advantages, for example, it does not require exosomal labeling and is easy to operate, which can reduce cross-contamination. However, the whole separation process consumes a lot of time (>4 h), with poor repeatability and stability. Many impurities are detected in the precipitates, such as subcellular organelles, virion, and co-purifying protein aggregates, which can influence the results of protein quantitation and mass spectrometry.^18^

## Density-gradient centrifugation

Density-gradient centrifugation is an improved version of the differential centrifugation approach for exosomal isolation by reducing the density from bottom to top. Density-gradient centrifugation employs two or more separation media with various densities, including iodixanol and sucrose.^19^ This method can improve the elimination of high-molecular-weight proteins and can be applied to characterize the extracellular vehicles isolated from body fluids, which consist of elevated protein aggregate levels. The density-gradient centrifugation is beneficial for obtaining high exosome purity. However, the sedimentation rate of exosomes can be reduced due to a high viscosity of sucrose solution, leading to an extended time.^20^

## Polymer precipitation

Polymer precipitation is an effective and rapid method for exosomal purification, which involves the incubation of samples and precipitating agents, followed by filtration or low-speed centrifugation. The reagents employed for polymer precipitation-based exosome isolation include polyethylene, protein organic solvent precipitation, acetate, and protamine, among which polyethylene is most used.^21^ This technique is easy to operate without the need for time-consuming procedures or complex devices, which is suitable for a large sample size, and can be combined with other separation methods. However, the exosomes isolated by this technique are vulnerable to contamination with virus particles or lipoproteins, which can adversely affect further analyses.^22^

## Immunoaffinity

Immunoaffinity can be used to separate and purify biological particles according to the antibody-antigen-specific reaction. Generally, exosomes have specific surface antigens that can be targeted by specific antibodies. The common biomarkers applied for exosomal isolation include the tetraspanins CD-9, -63, −81, −82, and −151.^23^ Up to now, immunomagnetic beads are more commonly used. The exosomes can be targeted by antibody-coated beads, to separate them from the unbound impurities under the magnetic force. The immunoaffinity approach has high isolation purity and strong specificity and can be used to isolate a specific subclass of exosomes.^24^ However, the exosomes isolated by immunoaffinity chromatography cannot be stored for long periods, and are not appropriate for large-scale separation of exosomes. However, some interfering proteins may be generated due to the non-specific matrix interference, which limits the application of this technique.

## Ultrafiltration

Ultrafiltration can be used to separate exosomes according to their molecular size. Exosomes are isolated by removing impurities via filtering membranes with molecular weight cut-off (MWCO) or different pore sizes. During filtration, larger particles are not able to pass through the membrane filter, retaining smaller particles.^25^ This method has the advantages of high enrichment efficiency and low cost, without affecting the activity of exosomes. On the other hand, the disadvantages are low purity and exosomal clogging on the filtering membrane surface, which can result in a low recovery rate.

## Size-exclusion chromatography

Another size-based isolation method is size-exclusion chromatography (gel filtration). After adding the samples to the porous beads-containing column, the larger particles cannot enter the gel pores, and the elution along the gaps between the mobile phase and porous gels is more rapid, retaining the small molecules in the gel pores, which are ultimately eluted by the mobile phase.^26^ This approach has several advantages, such as ease of operation, low cost, preserving initial exosome morphology and structure, and retrieving exosomes with uniform size. Nevertheless, they can be doped with other particles with an equal size, leading to a low purity.

## Gene regulation by lncRNAs

Generally, lncRNAs are classified into intronic lncRNAs, antisense lncRNAs, and intergenic lncRNAs, most of which are transcribed by Pol II, having 3′-end poly(A) tails, and 5′-end m7G caps.^27^ Similar to mRNA, after transcription and processing, most lncRNAs are translocated to the extracellular matrix. The lncRNAs exported from the nucleus can be sorted into mitochondria or exosomes. lncRNAs can interact with DNA/RNA/proteins to modulate gene expression at the level of post-transcriptional processing, transcription, and chromatin modification.^28^^,^^29^

## Chromatin modification

lncRNAs can regulate epigenetic alterations by recruiting chromatin remodeling complexes to target genomic loci. lncRNAs have been demonstrated to be related to DNA- or histone-modifying enzymes, including mixed lineage leukemia (MLL) family methyltransferases, polycomb repressive complex (PRC), polycomb group proteins (PcG), and histone methyltransferase G9a, thereby regulating gene expression in cis or trans.^30^^,^^31^ The core PRC2 complex contains the embryonic ectoderm development (EED), suppressor of zeste 12 (SUZ12), and enhancer of zeste homolog 2 (EZH2), which functions as a methyl transferase to catalyze H3 trimethylation on K27 (H3K27me3), leading to transcriptional repression and chromatin compaction.^32^^,^^33^ For instance, lncRNA hox transcript antisense intergenic RNA (HOTAIR) recruits PRC2 members (*e.g.*, SUZ12 and EZH2) to the target genes, which causes H3K27me3 and gene silencing, ultimately resulting in metastasis and poor prognosis in breast cancer.^34^

Besides, there is another example: X-chromosome-inactivation (XCI). XCI is one of the epigenetic mechanisms that ensure X-chromosome dosage compensation between cells of males (XY karyotype) and females (XX).^34^ There are some ways to explain this phenomenon. The transcriptional repressor SPEN is crucial for X-linked gene silencing, which binds to the X inactive-specific transcript (Xist) repeat A via its RNA recognition motif (RRM) domains and interacts through its Spen paralog and ortholog carboxyl-terminal (SPOC) domain with the nuclear receptor corepressor (NCoR)/silencing mediator of retinoic acid and thyroid hormone receptor (SMRT) to recruit/activate histone deacetylase 3 (HDAC3). HDAC3 is responsible for the removal of histone H3 and H4 acetylation at the promoters and enhancers of genes located on the future inactive X (Xi).^35^^,^^36^ SPEN also inhibits transcription through different mechanisms independently of HDAC3 such as recruiting PRC2, which trimethylates H3K27me3 and enhances gene silencing.^37^^,^^38^

## Transcription regulation

lncRNAs can mediate gene transcription in the nucleus via binding to and recruiting different transcription factors to the gene promoter, thereby promoting or inhibiting gene transcription. The lncRNA HOTAIR can promote angiogenesis after up-regulation, thus activating the transcription of vascular endothelial growth factor (VEGF) by targeting its promoter.^39^ The lncRNA Evf2 is transcribed from an ultraconserved distal enhancer and can recruit the transcription factor distal-less homeobox 2 (DLX2) for binding to this enhancer, thereby inducing the expression of adjacent protein-coding genes.^40^

## Post-transcriptional processing regulation

lncRNAs exhibit post-transcriptional regulation by sequestering miRNAs and interacting with splicing factors. The most common form of lncRNA-regulated miRNA expression is that lncRNAs can act as a molecular sponge to modulate mRNA expression. The lncRNA ZFAS1, an oncogene, promotes hepatocellular carcinoma metastasis by sponging miRNA-150, thus up-regulating zinc finger E-box binding homeobox 1 (ZEB1), matrix metallopeptidase 14 (MMP-14), and MMP-16.^41^ It has been reported that the lncRNA metastasis-associated lung adenocarcinoma transcript 1 (MALAT1) can affect the positioning of the SR family member of pre-mRNA splicing factors to the transcription site. SR proteins are multifunctional proteins that participate in pre-mRNA splicing. The lncRNA MALAT1 can induce serine and arginine-rich splicing factor 1 (SRSF1) to regulate its target cleavage, thereby promoting the formation of anti-apoptotic splicing isoforms and activating the mechanistic target of rapamycin (mTOR) pathway via the selective splicing of S6 kinase 1 (S6K1).^42^

## Exosomal lncRNAs in renal diseases

Exosomal lncRNAs are crucial to the abnormal development of several renal diseases. By interacting with three domains of SRSF1, such as the c-terminal domain and two RNA recognition motifs (RRM1 and RRM2), the exosomal lncRNA taurine up-regulated 1 (TUG1) derived from human urine-derived stem cells encourages the degradation of Acyl CoA synthetase long-chain family member 4 (ACSL4) mRNA.^43^^,^^44^ This decreases the expression of the corresponding proteins, which is a sensitive monitor of ferroptosis, and their down-regulation can attenuate cell ferroptosis, as a result of further improving ischemia–reperfusion injury-induced AKI.^43^^,^^45^^,^^46^ Liver cell-derived exosomal lncRNA-metallothionein 1D pseudogene (MT1DP) may change the expression or activities of its binding partners (*e.g.*, mRNA and protein), thereby enhancing the pro-apoptotic member Bcl-2-associated X (Bax) and reducing the anti-apoptotic member Bcl-xL via uncharted pathway, ultimately causing mitochondria-dependent kidney cell apoptosis.^47^ Researchers discovered 30 differentially expressed ncRNAs originating from urine exosomes that are promising biomarkers for early detection of CKD patients via ncRNASeqScan analysis. Eight antisense RNAs (PCBP1-AS1, EAF1-AS1, RP11-378E13.4, RP11-315I20.1, RP11-700F16.3, RP11-68I3.2, RP11–1382.1, and RP11-98D18.1) were varied in the exosomes produced from CKD patients compared with healthy controls. In eukaryotes, it has been demonstrated that antisense RNAs or long-intergenic noncoding RNAs (lincRNAs), which are produced in the opposite direction from the mRNA sense strands or the sense to main transcripts or hnRNAs, control the expression of genes.^48^ Exosomal lncRNAs also affect tumor progression, metastasis, and treatment resistance. lncRNA activated in renal cell carcinoma with sunitinib resistance (lncARSR) could be found in the exosomes derived from renal cell carcinoma. It is a competitive endogenous RNA of miR-34/miR449-5p that induces the transformation of macrophage phenotype from M1 to M2 via activation of the signal transducer and activator of transcription 3 (STAT3) pathway and promotes cytokine secretion, phagocytosis, and angiogenesis, thus significantly promoting the development of tumors.^49^ Furthermore, after competitively binding to miRNA-449 and miRNA-34, lncARSR promoted sunitinib resistance by up-regulating AXL/c-MET and activating ERK, AKT, and STAT3 signaling. In turn, the activated AKT increased the expression of lncARSR by inhibiting the transcription factors forkhead box protein O1 (FOXO1) and FOXO3a in sunitinib-resistant renal cell carcinoma cells. This created a positive feedback loop. Additionally, it was discovered that exosome-mediated secretion of lncARSR from resistant cells might convert sunitinib-sensitive cells into resistant cells and propagate drug resistance.^50^ In a hypoxic microenvironment, H3 histone-lysine-4 monomethylation (H3K4me1) was elevated in the regulatory elements of hypoxia-induced lncRNA (lncHILAR). Aberrant methylation and acetylation could dominate the up-regulated expression of lncHILAR in renal cell carcinoma cells. lncHILAR promotes renal cell carcinoma metastasis and invasion by acting as a competing endogenous RNA (ceRNA) for miR613/206/1-1-3p, which results in the up-regulated expression of C-X-C motif chemokine receptor 4 (CXCR4) and Jagged-1.^51^ The lncRNA differentiation-antagonizing non-protein-coding RNA (DANCR) is expressed in bone marrow mesenchymal stem cell-derived exosomes. By promoting E3 ubiquitin ligase SMURF2-mediated ubiquitylation and accelerating SIRT1 degradation, DANCR promotes CD4^+^ T cell differentiation into Treg cells, which induces immunological tolerance to kidney transplantation, increases patient survival, and improves the function of a transplanted kidney.^52^, ^53^, ^54^, ^55^

## Circulating lncRNAs in AKI and CKD

Additionally, there is still a sea of circulating lncRNAs that play significant roles in the pathogenesis of AKI and CKD.

## Sepsis-induced AKI

The harmful inflammatory cascade, a feature of sepsis, also leads to AKI. Compared with patients without AKI, those with sepsis that is worsened by AKI have a much higher fatality rate. Moreover, AKI patients with sepsis have a higher mortality rate compared with those with other etiologies.^56^ Here is a list of lncRNAs that have the potential to be diagnostic and therapeutic biomarkers (Table 1).

**Table 1:** lncRNAs in sepsis-induced acute kidney injury.

lncRNA	Expression level	Pathway	Reference
lncRNA PVT1	Up	miR-27a-3p/OXSR1	57
	Up	miR-17-5p NF-κB	58
	Up	miR-20a-5p/NLRP3	59
lncRNA NEAT1	Up	miR-22-3p/CXCL12/NF-κB	60
	Up	miR-93-5p/TXNIP	61
	Up	miR-125a-5p/TRAF6/TAK1	62
	Up	miR-22-3p/NF-κB	63
lncRNA MALAT1	Up	miRNA-135b-5p/NLRP3	64
	Up	miR-205	65
	Up	miR-370-3p/HMGB1	66
	Up	miR-146a/NF-κB	67
lncRNA SNHG14	Up	miR-495-3p/HIPK1	68
	Up	miR-93/IRAK4/NF-κB, miR-93/IL-6R/STAT3	69
lncRNA MEG3	Up	miR-18a-3p/GSDMD	70
	Up	miR-21/PDCD4	71
lncRNA CRNDE	Down	miR-181a-5p/PPARα	72
	Up	TLR3/NF-κB	73
lncRNA NORAD	Up	miR-577/GOLPH3	74
lncRNA GAS6-AS2	Up	miR-136-5p/OXSR1	75
lncRNA ZFAS1	Down	miR3723p/PPARα	76
lncRNA XIST	Down	miR-155-5p/WWC1	77
lncRNA KCNQ1OT1	Up	miR-212-3p/MAPK1/NF-κB	78
lncRNA NONRATG019935.2	Down	HuR/Tp53	79
lncRNA CASC2	Down	miR-545-3p/PPARα	80
lncRNA CASC9	Down	miR-424-5p/TXNIP	81
lncRNA NKILA	Up	miR-140-5p/CLDN2	82
lncRNA TapSAKI	Up	miR-205/IRF3	83
lncRNA RMRP	Up	miR-206/DDX5, NLRP3	84
lncRNA SIKIAT1	Up	miR-96/FOXA1	85
lncRNA CCAT1	Down	miRNA-155/SIRT1	86
lncRNA TCONS_00016406	Down	miR-687/PTEN	87
lncRNA HOXA-AS2	Down	miRNA-106b-5p/Wnt/β-catenin, NF-κB	88
lncRNA DANCR	Down	miR-214/KLF6	89
lncRNA TCONS_00016233	Up	miR-22-3p/AIFM1	90
lncRNA ANRIL	Up	miRNA-199a/TLR4/NF-κB	91
lncRNA TUG1	Down	Nrf2/HO-1	92
lncRNA SNHG5	Up	miR-374a-3p/TLR4/NF-κB	93
lncRNA RMRP	Up	ELAVL1/COX2	94

**Figure 2:**
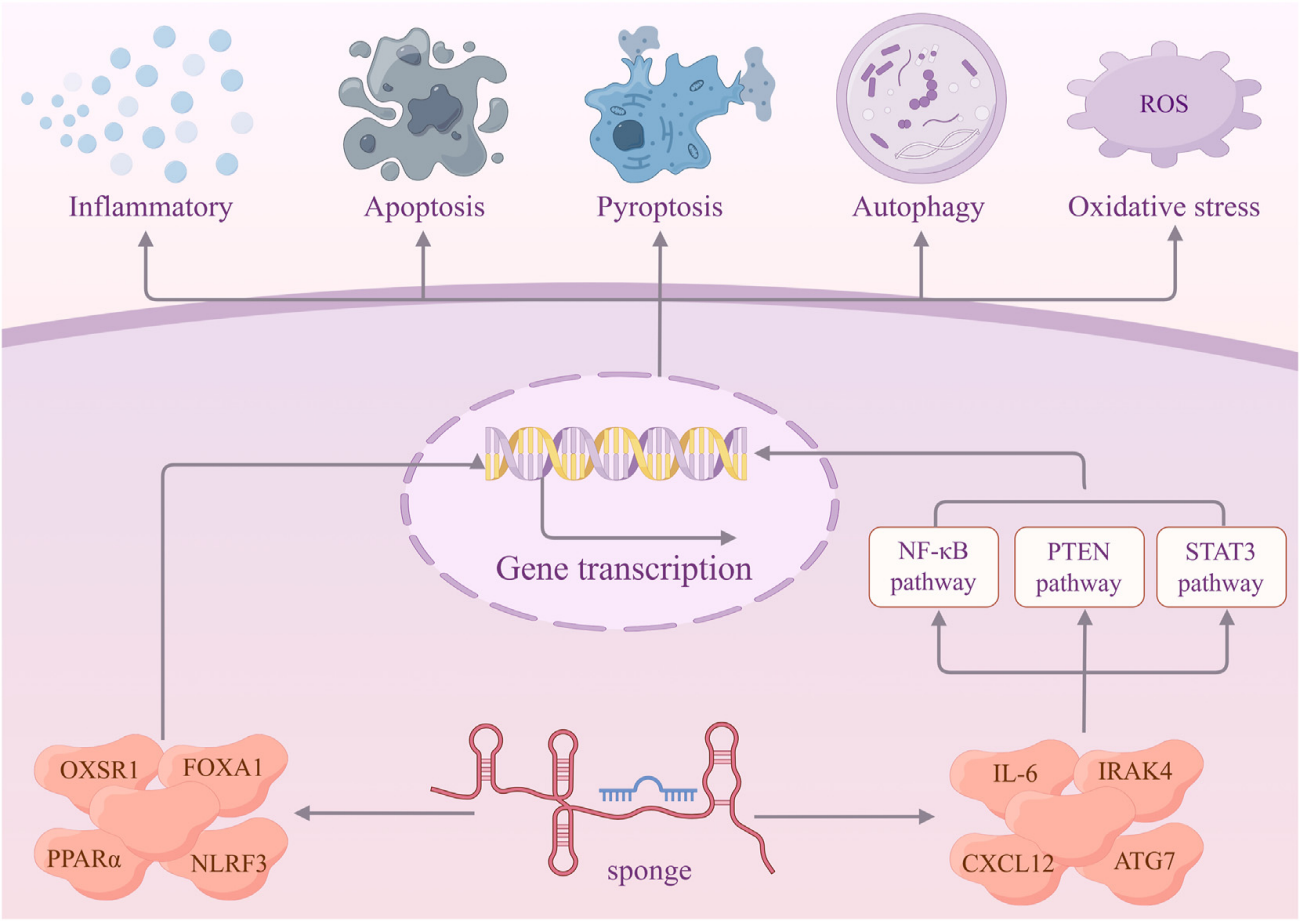
lncRNAs in sepsis-induced acute kidney injury (by Figdraw.com). lncRNAs function as miRNA sponges to positively or negatively regulate sepsis-induced acute kidney injury through specific signaling pathways.

LncRNAs can regulate sepsis-induced AKI through specific signaling pathways (Fig. 2). LncRNA nuclear-enriched abundant transcript 1 (NEAT1) was highly expressed in sepsis patients and lipopolysaccharide-induced kidney tubular epithelial cells (HK-2) or mouse macrophage cells. NEAT can directly interact with miRNA-22-3p, and this miRNA is a direct target of CXC chemokine ligand 12 (CXCL12) 3ʹ-UTR. After the expression of p-IκB-α and p-P65, it has been found that the NEAT1/miRNA-22-3p/CXCL12 axis can regulate lipopolysaccharide-induced HK2 cell damage via nuclear factor-κB (NF-κB) signal transduction. Finally, experimental data showed that the concentration of interleukin-1β (IL-1β), tumor-necrosis-factor-alpha (TNF-α), and interleukin-6 (IL-6) increased significantly, thereby accelerating inflammatory response and exacerbating sepsis-induced AKI.^60^^,^^63^ Additionally, NEAT1 acts as a sponge of miRNA-93-5p that directly targets thioredoxin-interacting protein (TXNIP) by complementary binding sites in TXNIP 3ʹ-UTR. The consequence is that NEAT1 up-regulation can decrease and increase the protein expression of Bcl-2 and Bax, respectively, thus resulting in cell injury.^61^ What's more, NEAT1 regulates macrophage polarization by suppressing miRNA-125a-5p expression, promoting the expression of TNF receptor-associated factor 6 (TRAF6), a pivotal component of the toll-like receptor (TLR)-induced NF-κB signal transduction in immune responses, and transforming growth factor-activated kinase 1 (TAK1) protein phosphorylation, which enhances lipopolysaccharide-induced inflammatory responses via macrophage M1 polarization.^62^^,^^95^^,^^96^

Upon activation of the TLR4/NF-κB pathway, small nucleolar RNA host gene 14 (SNHG14) expression was up-regulated in lipopolysaccharide-induced HK-2 cells. SNHG14 promotes lipopolysaccharide-induced HK-2 cell apoptosis, oxidative stress, and inflammation by interacting with miRNA-93. Luciferase reporter assay was utilized to analyze the specific binding sites of miRNA-93 in the 3ʹ-UTR of IL-6R and IL-1 receptor-associated kinase 4 (IRAK4). SNHG14/miRNA-93 can activate STAT3 and NF-κB pathways by regulating IRAK4, a significant factor in IL-1*β*/NF-κB signaling, and IL-6R.^69^ *In vivo*, SNHG14 expression was increased in the plasma of AKI patients with sepsis. SNHG14, as a ceRNA, has complementary binding sequences targeting miRNA-495-3p. miRNA-495-3p has a putative binding sequence targeting the 3ʹ-UTR of homeodomain-interacting protein kinase 1 (HIPK1). SNHG14/miRNA-495-3p/HIPK1 interaction network increases and decreases the expression of Bax/cleaved-caspase 3 and Bcl-2, respectively, and promotes cell apoptosis.^68^

The expression of lncRNA maternally expressed gene 3 (MEG3) is increased in kidney tissues and cell models exposed to lipopolysaccharide. To induce the pyroptosis of tubular epithelial cells, MEG3 may directly bind to miRNA-18a-3p, and this miRNA links to the pyroptosis of gasdermin D (GSDMD).^70^^,^^97^ On the other hand, MEG3 serves as a miRNA-21 competitor endogenous RNA in TKPTS cells. The fact that the validated miRNA-21 target protein, programmed cell death protein 4 (PDCD4), is highly expressed in the damaged kidney tissues and lipopolysaccharide-stimulated TKPTS cells suggests that MEG3 can cause TKPTS cell apoptosis by sponging miRNA-21.^71^

Researchers have found that 881 and 332 lncRNAs were highly expressed in septic AKI or non-AKI compared with healthy controls, respectively.^90^ Among these, TCONS_00016233 is the top co-upregulated lncRNA (218- and 98-fold changes in septic AKI and non-AKI versus controls, respectively) via TLR4/p38MAPK axis. The 3′ sequence of TCONS_00016233 consisted of the miRNA-22-3p binding site, thereby inhibiting the activity and expression of miRNA-22-3p. Apoptosis-inducing factor mitochondrion-associated 1 (AIFM1) might be a direct target of miRNA-22-3p, which increases the expression of TNF-α, IL-1β, and cleaved caspase-3.^90^ lncRNA NONRATG019935.2 indirectly reduced the mRNA stability of Tp53 by disrupting the combination of human antigen R (HuR) and Tp53 3ʹ-UTR, thus restraining the p53-mediated renal tubular epithelial cell apoptosis.^79^ Sepsis-induced kidney injury-associated transcript 1 (SIKIAT1) is highly expressed in sepsis patients and lipopolysaccharide-stimulated HK-2 cells, and functions as a sponge for miRNA-96-3p to induce forkhead box A1 (FOXA1) expression and promote apoptosis.^85^ lncRNA potassium voltage-gated channel subfamily Q member 1 opposite strand/antisense transcript 1 (KCNQ1OT1) directly interacts with miRNA-212-3p, and this miRNA directly targets mitogen-activated protein kinase 1 (MAPK1) 3ʹ-UTR and negatively modulates MAPK1 in lipopolysaccharide-stimulated HK-2 cells, inducing the protein levels of p–NF–κB and p-p38MAPK and enhancing NF-κB nuclear translocation.^78^ The expression of colorectal neoplasia differentially expressed (CRNDE) is down-regulated in the kidneys of US rats and miRNA-181a-5p is a downstream effector of CRNDE. miRNA-181a-5p directly targets the 3ʹ-UTR of peroxisome proliferator-activated receptor-α (PPARα) and represses its protein level, while Bax and Bcl-2 appear to be up-regulated and down-regulated, respectively.^72^ lncRNA TCONS_00016406 is down-regulated in the kidneys of AKI mouse and HK-2 cell models. lncRNA TCONS_00016406 can bind to miRNA-687 and phosphatase and tensin homolog deleted on chromosome 10 (PTEN) and is responsible for the pathogenesis of AKI by mediating inflammation and apoptosis. The protein levels of Bax and Bcl-2 were remarkably increased and decreased, respectively.^87^ Non-coding RNA activated by DNA damage (NORAD) was overexpressed in AKI and could down-regulate miRNA-577. Golgi phosphoprotein 3 (GOLPH3) was a downstream gene of miRNA-577 and was highly expressed in AKI patients with sepsis. The protein levels of Bax and Bcl-2 were increased and decreased, respectively, thereby enhancing HK-2 cell apoptosis and inflammation.^74^

## Ischemia–reperfusion injury

Renal ischemia–reperfusion injury is a severe medical condition that leads to AKI, resulting in renal dysfunction and a high mortality rate. Ischemia–reperfusion injury has been commonly reported during cardiovascular and urologic surgery, kidney transplantation, trauma, and shock, and there have been no effective treatments until now.^98^ However, the current studies of lncRNAs could provide new ideas for diagnosis and treatment (Table 2).

**Table 2:** lncRNAs in ischemia–reperfusion injury.

lncRNA	Expression level	Pathway	Reference
lncRNA H19	Up	miRNA-30a-5p/Dll4, ATG5, Snai1	99
	Up	miR-130a/BCL2L11	100
lncRNA MEG3	Up	miR-129-5p/HMGB1	101
	Up	miR-145-5p/RTKN/Wnt/β-catenin/c-MYC	102
lncRNA TUG1	Down	miR-494-3p/E-cadherin	103
	Up	miR-29a/PTEN	104
lncRNA HCG18	Up	miR-16-5p/Bcl-2	105
lncRNA ENSMUST_147219	Up	miR-221-5p/IRF6	106
lncRNA 121686	Up	miR-328-5p/HtrA3	107
lncRNA 122049	Up	miR-330-5p/ELK1	108
lncRNA SNHG14	Up	miR-124-3p/MMP2	109
lncRNA XIST	Up	miR-142-5p/PDCD4	110
LINC00963	Up	miR-128-3p/JAK2/STAT1	111
lncRNA GAS5	Up	miR-21/TSP-1	112
LINC00520	Up	miR-27b-3p/OSMR PI3K/AKT	113
lncRNA IRAR	Up	C/EBP β/lncRNA IRAR/CCL2, CXCL1, CXCL2	114
lncRNA 136131	Up	miR-378a-3p/Rab10	115
lncRNA 148400	Up	miR-10b-3p/GRK4	116
lncRNA ENSMUST00000171502	Up	miR-130b-3p/Mybl-1	117

**Figure 3:**
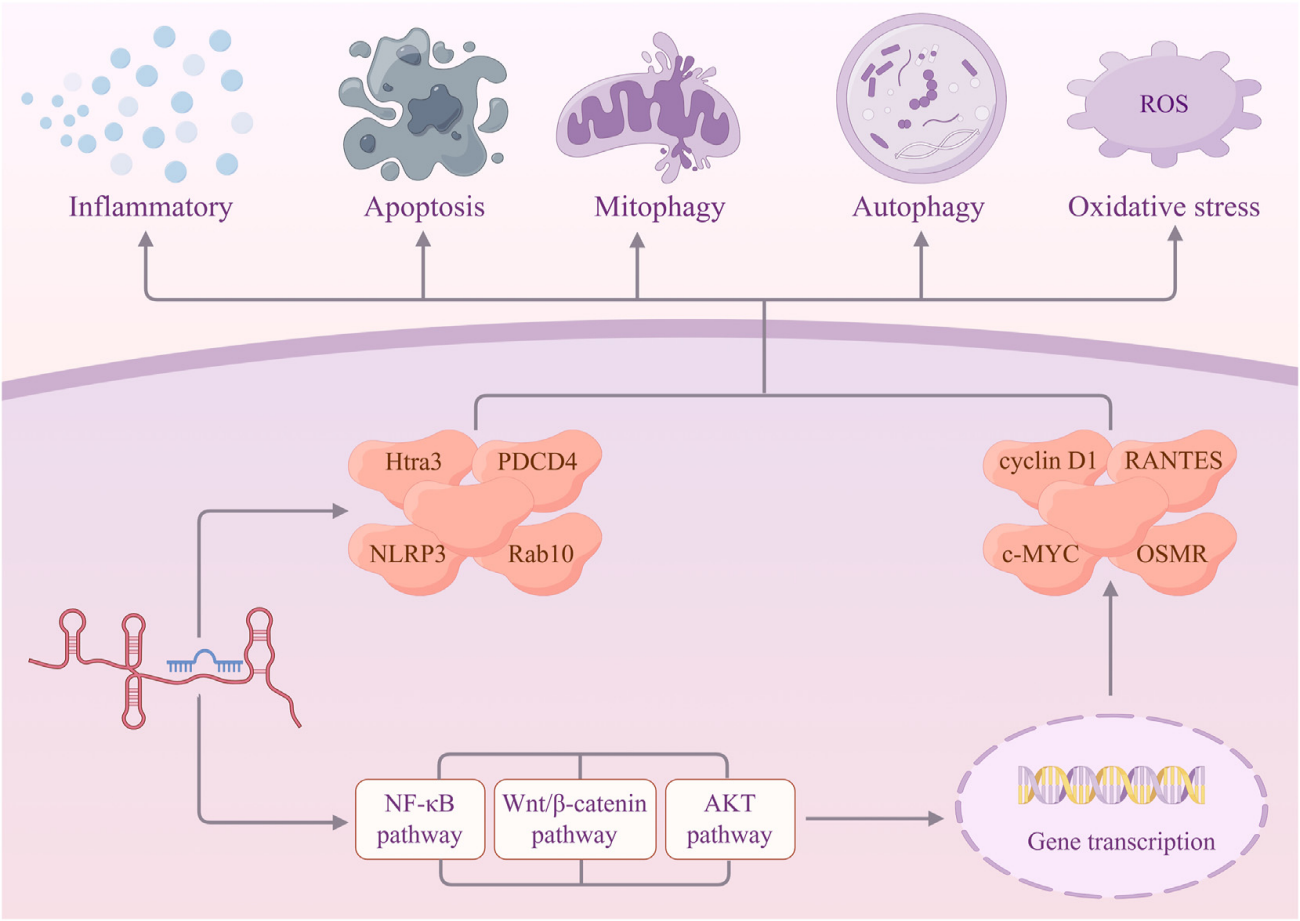
lncRNAs in ischemia–reperfusion injury-induced acute kidney injury (by Figdraw.com). lncRNAs act as miRNA sponges to regulate ischemia–reperfusion injury-induced acute kidney injury positively or negatively through certain signaling pathways.

The following figure illustrates the pattern of lncRNA regulation of IRI-induced AKI (Fig. 3). LncRNA H19 modulates the expression of miRNA-130a, and this miRNA targets BCL2L11 gene in HEK-293 cells, thereby inducing cell apoptosis.^100^ MEG3 could up-regulate rhotekin (RTKN) expression by binding to miRNA-145-5p, thus inducing HK-2 cell mitophagy and apoptosis after ischemia–reperfusion injury. MEG3 activates the Wnt/β-catenin pathway by up-regulating RTKN expression. c-MYC, as a downstream effector of the Wnt/β-catenin pathway, can serve as a transcription factor for MEG3 activation.^102^ lncRNA ENSMUST_147219 acts as a ceRNA to sponge miRNA-221-5p and subsequently increases the expression of interferon regulatory factor 6 (IRF6), which in turn up-regulates the expression of cleaved caspase-3, thus promoting the development of ischemic AKI.^106^ Mmu-lncRNA 121686 and hsa-lncRNA 520657 directly sponge miRNA-328-5p, which binds to the sequence of 3′-UTR of high-temperature requirement factor A 3 (Htra3), causing HK-2 and BUMPT cell apoptosis as well as caspase-3 activation. About the upstream regulatory mechanisms of hsa-lncRNA 520657 and mmu-lncRNA 121686, methyltransferase 3 (METTL3) interacts with hsa-lncRNA 520657 and mmu-lncRNA 121686 n6-methyladenosine (m^6^A) sites, thereby affecting their stability.^107^ Reversible m^6^A RNA modification alters the stability and functionality of RNA. lncRNA can have its spatial structure and stability changed by the m^6^A alteration to regulate its ability to attach to other RNA or proteins.^118^ Transcription factor CCAAT enhancer-binding protein beta (C/EBP-β) can bind to the lncRNA IRAR promoter directly; IRAR overexpression induces C–C motif chemokine ligand 2 (CCL2), CXCL1, CXCL2 expression, promotes IL-6 secretion, and induces cell apoptosis.^114^ lncRNA 122049 can directly sponge miRNA-330-5p, and subsequently increase the expression of ELK1 to reduce kidney cell apoptosis.^108^

lncRNA growth arrest-specific 5 (GAS5) acts as a ceRNA for miRNA-21 and this miRNA interacts with the 3′-UTR of thrombospondin-1 (TSP-1), thereby inducing apoptosis during renal ischemia–reperfusion injury.^112^ The expression levels of OSMR and LINC00520 are increased in AKI renal tissues, whereas miRNA-27b-3p is decreased in AKI renal tissues, and there are potential binding sites at the 3ʹ- and 5ʹ-UTR of LINC00520-OSMR-miRNA-27b-3p.^113^

## Nephrotoxic agents

Cis-diamminedichloroplatinum (II)-induced nephrotoxicity can lead to irreversible kidney injury related to high mortality and morbidity. High levels of lnc-MEG3 in this kind of nephrotoxicity were observed in both cell and animal models and consequently inhibited the expression of miRNA-126. Down-regulation of miRNA-126 can activate the AKT/tuberous sclerosis complex (TSC) pathway, thereby suppressing mTOR-regulated autophagy, inflammation, apoptosis, and kidney cell damage.^119^ The expression of lncRNA opa-interacting protein 5 antisense RNA 1 (OIP5-AS1) was remarkably decreased in cisplatin-induced HK-2 cells and AKI mouse models. OIP5-AS1 acts as a sponge of miRNA-144-5p, and this miRNA is a direct target of pyruvate kinase M2 (PKM2). PKM2 overexpression could markedly reverse the effect of miRNA-144-5p mimics on cisplatin-induced HK-2 cell damage.^120^ The expression level of prostate cancer non-coding RNA 1 (PRNCR1) was dramatically reduced in cisplatin-stimulated AKI mice. miRNA-182-5p was negatively modulated by PRNCR1 and could up-regulate the expression of enhancer of zeste homolog 1 (EZH1), thus inducing apoptosis.^121^

## Chronic kidney disease

According to mounting evidence, AKI is a key contributor to CKD, and recurrent AKI in CKD patients may hasten its progression to end-stage renal disease.^122^ AKI-to-CKD had a pooled adjusted hazard ratio of 4.3 compared with CKD without a history of AKI, according to the results of prior research.^123^ A total of 425 differentially expressed lncRNAs were associated with CKD in the 2022 bioinformatics analysis, among which 196 were up-regulated and 229 were down-regulated when compared with healthy individuals. The lncRNAs lnc-IFITM2-3 and lnc-FBXL5-7 can serve as diagnostic biomarkers for CKD.^124^

## Diabetic nephropathy

**Figure 4:**
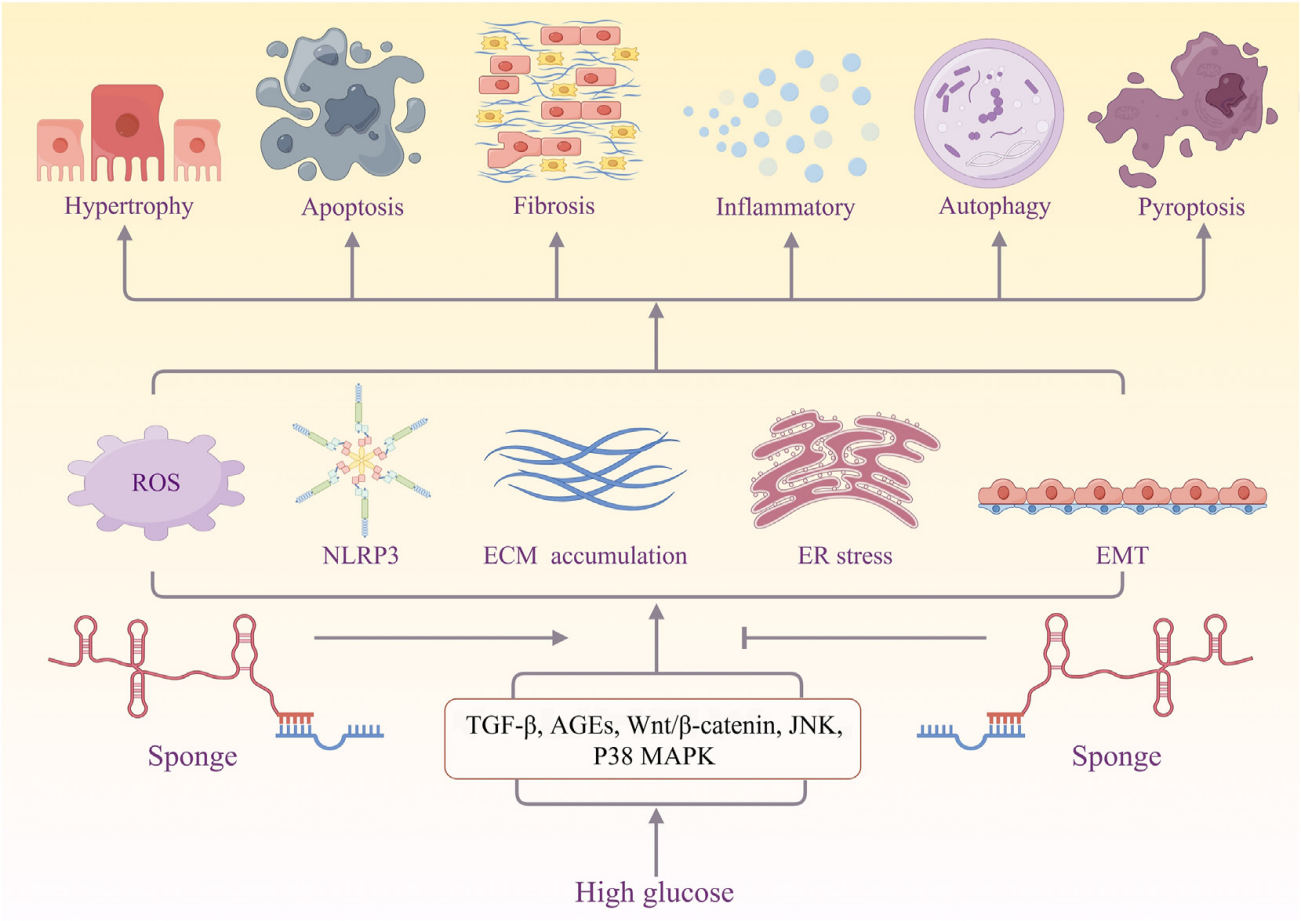
lncRNAs in diabetic nephropathy (by Figdraw.com). Under hyperglycemic conditions, certain pathways lead to extracellular matrix (ECM) buildup, epithelial-mesenchymal-transition (EMT), endoplasmic reticulum (ER) stress, oxidative stress, fibrosis, and inflammatory responses. lncRNAs, as miRNA sponges, influence these pathogenic processes positively or negatively, contributing to diabetic kidney complications such as apoptosis, autophagy, pyroptosis, hypertrophy, fibrosis, and inflammation.

Over the last three decades, diabetic nephropathy (DN) has witnessed a surge, emerging as a prevalent microvascular complication and a leading cause of CKD globally. DN can lead to irreversible renal function loss, eventually progressing to end-stage kidney disease. LncRNAs, acting as miRNA sponges, influence pathogenic processes that contribute to diabetic kidney complications (Fig. 4). In urinary extracellular vesicles from DN samples, a total of 1684 differentially expressed mRNAs, 126 differentially expressed lncRNAs, 123 differentially expressed circRNAs, and 66 differentially expressed miRNAs were identified. Following the analyses, two lncRNAs, namely LINC000958 and PVT1, were identified and selected from urinary extracellular vesicles as potential biomarkers for DN.^125^ In individuals with DN, the increased presence of LINC00355 in podocytes correlated with a deterioration in renal function. LINC00355, operating biologically in the nucleus, binds to EZH1, epigenetically targeting catenin beta interacting protein 1 (CTNNBIP1) by repressing H3K4 trimethylation. This, in turn, triggers endoplasmic reticulum stress and Wnt/β-catenin signaling. Simultaneously, LINC00355 regulates endoplasmic reticulum stress-induced podocyte damage in DN by attracting EZH1 to the promoter region of CTNNBIP1.^126^ Elevated levels of thioredoxin-interacting protein (TXNIP)-induced pyroptosis contribute to DN pathology. The cytoplasmic Prader Willi/Angelman region RNA, SNRPN neighbor (PWARSN), functions as a sponge for miR-372-3p, enhancing TXNIP expression. Additionally, nuclear PWARSN interacts and promotes the degradation of RNA binding motif protein X-linked (RBMX) through ubiquitination, leading to a reduction in H3K9me3 enrichment at the TXNIP promoter. This results in the initiation of TXNIP transcription, activating the nod-like receptor protein-3 (NLRP3) inflammasome and causing cell pyroptosis.^127^ Through the miR-9/sirtuin 1 (SIRT1) axis, the lncRNA TUG1 protects podocytes from high glucose-induced apoptosis and mitochondrial dysfunction.^128^ In the development of DN, TTN-AS1 increases extracellular matrix accumulation through the miR-493-3p/forkhead box P2 (FOXP2) axis. The findings of this study might offer potential therapeutic options for DN in a different way.^129^ Hyperglycemia represses miR-214-3p by inducing lncRNA-CES1P1, which promotes the expression of the inflammatory factors IL-17, IκB, NF-κB, and IL-6, ultimately leading to the development of DN.^130^ Through up-regulating the levels of NLRP3, gasdermin-N domain (GSDMD-N), caspase 1, fibronectin, collagen I/III, IL-1β, and IL-18, the lncRNA ZFAS1 facilitates fibrosis and scortosis in HK-2 cells.^131^ lncRNA UCA1 acts as an anti-pro-cytokine by directly inhibiting the expression of miR-206, thereby inhibiting apoptosis and inflammation of renal tubular epithelial cells.^132^ Moreover, through the MEG3/miR-21/ORAI1 axis, lncRNA MEG3 prevents renal fibrinoid necrosis in DN.^133^ lncRNA Miat increases Sox4 expression, regulating p53 ubiquitination and acetylation, inhibiting downstream factors CyclinB/cdc2 by enhancing p21cip1/waf1 activity. Miat interacts with Sox4 by sponging miR-130b-3p, effectively enhancing glomerular podocyte injury and mitotic dysfunction, ultimately exacerbating proteinuria.^134^

## Lupus erythematosus nephritis

**Figure 5:**
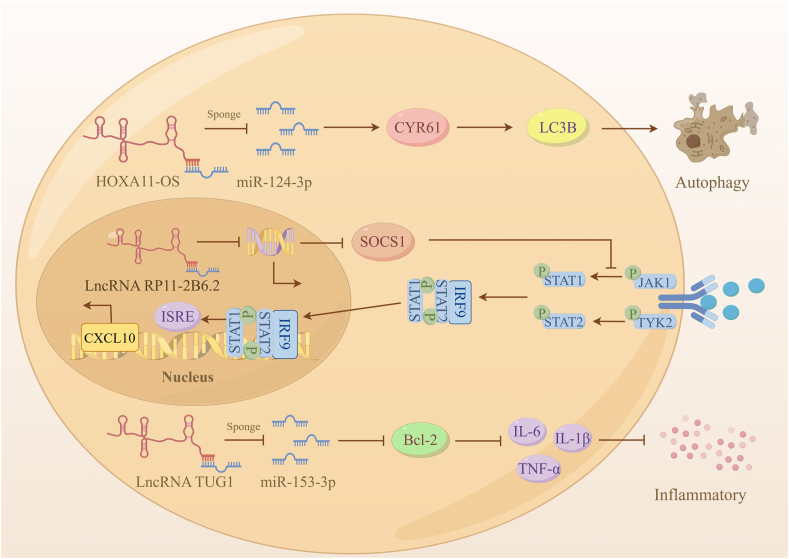
lncRNAs in lupus nephritis (by Figdraw.com). HOXA11-OS acts as a competitive endogenous RNA of miR-124-3p, promoting Cyr61 production and exacerbating podocyte damage through enhanced autophagy. lncRNA RP11-2B6.2 enhances IFN-I-induced phosphorylation of TYK2, JAK1, and STAT1 by suppressing SOCS1 at the transcriptional level, leading to increased expression of IFN-I-induced inflammatory genes. lncRNA TUG1 blocks lupus nephritis progression by inhibiting apoptosis and inflammatory responses through the miR-153-3p/Bcl-2 axis.

Systemic lupus erythematosus is a chronic autoimmune disease, and one of its most severe organ manifestations is lupus nephritis, which can eventually progress to end-stage renal disease. In a transcriptomic analysis of mouse kidneys with lupus nephritis, 11 differentially expressed lncRNAs were identified. These lncRNAs targeted and co-expressed with six immune and systemic lupus erythematosus-associated genes. Notably, lncRNA Gm20513 was found to positively regulate the expression of the systemic lupus erythematosus-associated gene H2-Aa.^135^ Additionally, an analysis of lncRNAs packaged into exosomes by sequencing revealed four lncRNAs (LINC01015, LINC01986, AC087257.1, and AC022596.1) within the network organization. These lncRNAs targeted critical pathways implicated in inflammation, fibrosis, epithelial–mesenchymal transition, and actin cytoskeleton.^136^ The opposite strand of homeobox A11 (HOXA11-OS) functioned as a competitive endogenous RNA of miR-124-3p, promoting the production of cysteine-rich 61 (Cyr61) and exacerbating podocyte damage by boosting the autophagy. HOXA11-OS knockdown produced the opposite result.^137^ lncRNA TUG1 was identified as a factor blocking lupus nephritis progression by inhibiting apoptosis and inflammatory responses through the miR-153-3p/Bcl-2 axis.^138^ Furthermore, lncRNA RP11-2B6.2 was found to inhibit SOCS1 expression by reducing chromatin accessibility upstream of SOCS1 gene and inhibiting the promoter activity of SOCS1 gene. Inadequate induction of SOCS1 led to kidney damage via enhanced interferon responses in mice. This discovery provides a new therapeutic target to alleviate over-activated type I interferon (IFN–I) signaling in lupus nephritis.^139^ Figure 5 illustrates the regulatory mechanism by which the aforementioned three lncRNAs operate.

## Renal fibrosis

A common pathological manifestation of chronic kidney disease is fibrosis, as characterized by loss of kidney function, a key factor in progression to chronic renal failure.^140^ Fat mass and obesity-associated protein (FTO) could suppress lncRNA GAS5 expression by attenuating m^6^A modification of lncRNA GAS5, enhancing transforming growth factor β1 (TGF-β1)-induced levels of epithelial-mesenchymal-transition-related proteins (N-cadherin, Snail, and Vimentin) and inflammatory cytokines (TNF-α, IL-1β, and IL-6) in HK-2 cells, and promoting epithelial-mesenchymal-transition process and inflammation responses.^141^ Additionally, it has been reported that the primary methyltransferase of m^6^A modification on lncRNA MALAT1 is m^6^A methyltransferase-like 3 (METTL3). MALAT1 has seven m^6^A sites, and the overexpression of MALAT1 in kidney fibrosis is a result of m^6^A alteration. MALAT1 can act as a sponge to miRNA-145 in HK2 cells exposed to TGF-β1 and then miRNA-145 regulates FAK, inducing epithelial-mesenchymal-transition and extracellular-matrix protein deposition, and promoting the proliferation, migration, and viability of HK2 cells.^142^ Also, lncRNA GAS5 inhibited miRNA-21 expression in a direct and mechanistic manner. It subsequently down-regulated the expression of MMP-2 and MMP-9 (the miRNA-21 downstream target genes), which led to extracellular matrix synthesis and accumulation.^143^ lncRNA Neat1_2 can bind to miRNA-129-5p and prevent this miRNA from reducing the levels of fas-associated protein with death domain (FADD), caspase-8, and caspase-3, and ultimately facilitates tubular epithelial cell apoptosis.^144^ Besides, miRNA-129 is directly targeted by Neat1, which binds to collagen type I. Down-regulated expression of miRNA-129 could reverse NEAT1 silencing-induced inhibition of kidney fibrosis.^145^ By specifically targeting miRNA-124-3p, lncRNA KCNQ1OT1 knockdown has an anti-fibrotic impact by reducing cell proliferation and the production of α-smooth muscle actin (α-SMA) and fibronectin.^146^ The down-regulation of miRNA-615 and attenuation of its inhibitory effect on hepatocyte nuclear factor-1β (HNF-1) caused by the overexpression of lncRNA4474 may delay the course of renal fibrosis, and miRNA-615 could activate the Wnt pathway by suppressing HNF-1.^147^ lncRNA ENST00000453774.1 binds to miRNA-324-3p and negatively regulates its expression, which promotes theneuregulin-1 (NRG1) expression and inactivates PI3K/AKT signal transduction, thus inducing cell autophagy and suppressing kidney fibrosis.^148^ lncRNA XIST and miRNA-19b had a direct interaction, and miRNA-19b was bound to SRY-box 6 (SOX6). Through the down-regulation of SOX6 by miRNA-19b, XIST knockdown prevented apoptosis, inflammation, and fibrosis.^149^ The WISP1/PKB signal transduction may be activated by lncRNA Gm12840 to function as a sponge for miRNA-677-5p to regulate fibroblast activation triggered by TGF-β1.^150^ Overexpression of lncRNA ENST00000453774.1 could promote reactive oxygen species defense via nuclear factor erythroid 2-related factor (Nrf2)-keap1/heme oxygenase 1 (HO-1)/NAD (P)H: quinone oxidoreductase 1 (NQO-1) signaling by inducing prosurvival autophagy, and then reduce extracellular matrix-related proteins (*e.g.*, collagen I and fibronectin), which can be a new anti-fibrotic treatment for kidney diseases.^151^

**Figure 6:**
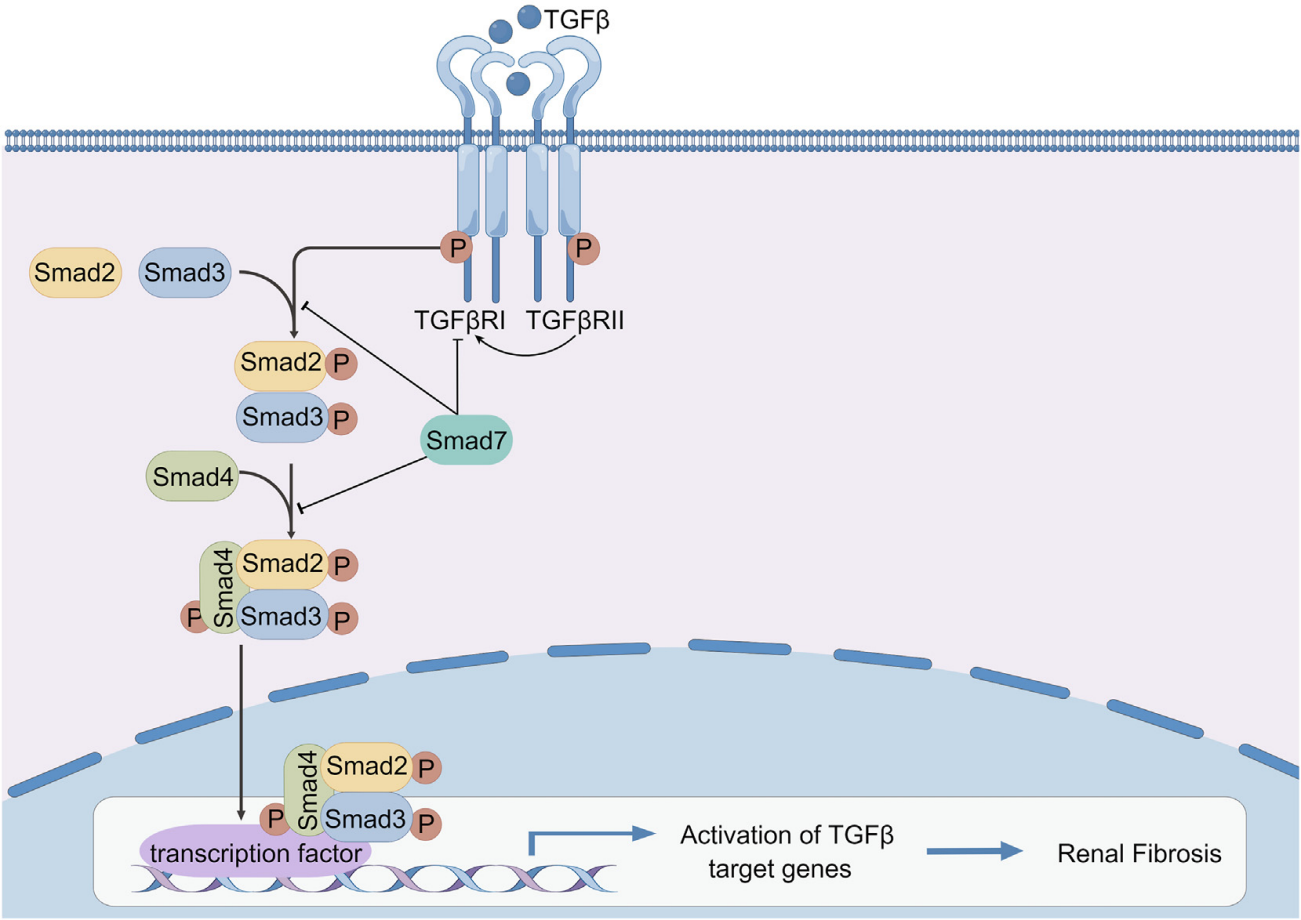
Smad3 signaling pathways in renal fibrosis (by Figdraw.com). TGF-β first binds to TβRII, which is present in the cell membrane in an oligomeric form with intrinsic kinase activity. TβRI is phosphorylated by binding to TβRII, leading to the activation of its kinase activity and subsequent recognition of bound Smad2/3, which phosphorylates it. Smad2/3 then binds to Smad4, forming the Smad complex, which translocates into the nucleus and regulates the target gene transcription.

Smad signaling has been identified as a crucial pathway in TGF-β1-mediated kidney fibrosis (Fig. 6). TGF-β first binds to the type–II–receptor (TβRII), which is present in the cell membrane with intrinsic kinase activity in an oligomeric form.^152^ TβRI is phosphorylated by binding to TβRII, leading to activation of its kinase activity and subsequent recognition of bound Smad2/3, which phosphorylates it. Smad2/3 then binds to Smad4, forming the Smad complex, which can translocate into the nucleus and modulate the target gene transcription.^153^, ^154^, ^155^ Overexpression of Smad7 inhibits the TGF-β signaling pathway by inhibiting Smad2/3 activation, blocking complex formation, and ubiquitinating TβRI degradation, while it stimulates the production of IκBα to inhibit NF-κB pathway.^156^, ^157^, ^158^ A total of 21 lncRNAs for TGF-/Smad3 signaling in the kidney have been discovered using RNA sequencing and sequence analysis, two of which are involved in TGF-regulated kidney fibrosis (lncRNA np_5318 and lncRNA np_17856).^159^ lncRNA Erbb4-IR is induced by TGF-β1 through a Smad3-dependent mechanism and binds to the 3ʹ-UTR of Smad7 to inhibit the negative effect of Smad7 on TGF-β/Smad pathway, thus promoting the development of renal fibrosis.^160^ GF-β/Smad3-interacting lncRNA (lnc-TSI) reduces kidney fibrosis by negatively modulating the TGF-β/Smad pathway via binding to the MH2 structural domain of Smad3, disrupting the interaction between Smad3 and TGF-β receptor I, and inhibiting fibrosis-related downstream gene expression.^161^

## Others

The kidney is a major target organ affected by hypertension, and globally, the prevalence of hypertension-induced chronic nephropathy exceeds 23.6 million, significantly impacting patients' survival time.^162^ Through the analysis of data from the NCBI Gene Expression Omnibus (GEO), 20 differentially expressed lncRNAs were identified. In the ceRNA network, LINC00470 and SNHG14 emerged as hub nodes in the regulatory network, targeting a greater number of microRNAs and mRNAs. A ceRNA regulatory network directly associated with hypertensive nephropathy was established, and the insulin signaling pathway was identified as directly linked to hypertensive nephropathy, modulated by lncRNAs SNHG14, TUG1, ZNF252P-AS1, and MIR503HG.^163^ Autosomal dominant polycystic kidney disease (ADPKD), the most common inherited cause of chronic kidney disease with polycystin (PKD) 1 and 2 gene mutations, was investigated. Telomeric RNAs associated with telomeric repeats (TERRAs) engaged in the R-loop and were found to have higher expression and shorter telomere length in ADPKD patients with rapid disease progression. Intrafamilial variation in telomere length and TERRA levels with the same mutation suggested reliable epigenetic potential biomarkers for disease monitoring.^164^ In adenine-induced CKD, the expression of lncRNA SNHG-7, Beclin-1, and LC3-II is significantly down-regulated, and mir-34a and P62 expression are significantly up-regulated. This down-regulation actively inhibits the autophagy pathway, leading to increased renal cell inflammation and apoptosis.^165^

## Applications of lncRNAs

The evidence presented above clearly demonstrates the potential of lncRNAs to develop into novel molecules for disease diagnostics and therapy.

## lncRNAs as diagnostic biomarkers

At present, the most widely used clinical indicators for the diagnosis of AKI are blood urea nitrogen and serum creatinine. However, the response of creatinine to AKI is often delayed by 26–36 h.^166^ Therefore, the discovery of sensitive early diagnostic indicators is crucial and urgent. Thirty-one significantly dysregulated lncRNAs were identified from 38 studies by meta-analysis. lncRNAs XIST, transcript predicting survival in AKI (TapSAKI), MALAT1, cancer susceptibility candidate 2 (CASC2), and homeobox A cluster antisense RNA 2 (HOXA-AS2) are identified as potential therapeutic targets and predictive biomarkers for AKI.^167^ Renal lncRNA expression can be altered by aging and ischemia–reperfusion injury alike. In the control kidneys of aged mice, AK082072 was elevated, whereas RepA and H19 transcripts were reduced. Igf2as, Miat, SNHG6, SNHG5, Malat1, RNCR3, Neat1 v1, Linc1633, and Air were all elevated by ischemia–reperfusion injury, whilst Linc1242 was down-regulated. Aging and ischemia–reperfusion injury have an impact on the lncRNAs AK082072, H19, Six3os, and RepA transcript, indicating that lncRNAs play a diverse and intricate function in aging and renal damage.^168^ The lncRNA NEAT1 is found to be up-regulated in both blood and urine samples of patients with type 2 diabetes, it plays a significant role in initiating and advancing DN by modulating the expression and activity of microRNAs. NEAT1 shows promise as a potential biomarker for the early diagnosis of DN.^169^ Furthermore, increased expression of antisense non-coding RNA in the INK4 locus (ANRIL) in peripheral blood has been associated with the progression of DN. The sensitivity and specificity of ANRIL in diagnosing DN were measured at 83.3% and 90.5%, respectively, with an area under the curve for DN identification reaching 0.922. The circulation of ANRIL demonstrates robust predictive value for diagnosing DN and is identified as an independent risk factor for the disease.^170^ However, there are currently no established clinical practices for lncRNA application, and lncRNAs in preclinical studies need to be verified and approved by clinical trials. The early diagnosis of AKI would benefit immensely from the efforts to locate and characterize lncRNAs that may be utilized for a more accurate diagnosis of kidney diseases.

## lncRNAs as therapeutic targets

lncRNAs can contribute to the process of kidney injury. If certain lncRNAs are responsible for the development of kidney diseases, therapeutic strategies that target these molecules may help to ameliorate this pathological alteration. Targeting lncRNAs can be achieved using a variety of approaches, including small interfering RNAs (siRNAs), antisense oligonucleotide (ASO), small-molecule inhibitors, clustered regularly interspaced short palindromic repeats (CRISPR), and plant-derived natural compounds.^65^^,^^104^^,^^171^, ^172^, ^173^, ^174^, ^175^ siRNA is similar to ASOs in design. siRNA is a type of double-stranded antisense RNA that enters an organism and unspins into single-stranded RNA. This single-stranded RNA then binds to proteins for the formation of RNA-induced silencing complex (RISC), which is found in the cytoplasm and binds to complementary siRNA-targeted lncRNA to cause its degradation.^176^^,^^177^ ASO is a chemically synthesized single-stranded nucleotide molecule that binds to the complementary RNA and recruits RNase H located in the nucleus, thereby causing RNA degradation and affecting downstream protein expression.^178^ Thus, ASO and siRNA can effectively suppress nuclear and cytoplasmic lncRNAs, respectively. The CRISPR/Cas9 system contains Cas9 enzyme and single-guide RNA (sgRNA). sgRNAs guide the Cas9 nucleases to specific sites in the genome through complementary base pairing, and Cas9 enzymes cleave DNA sequences followed by protospacer-adjacent motifs.^179^^,^^180^ Direct deletion of the DNA sequences that produce lncRNAs via transcription is one of the two most often used methods of knockdown, as is the introduction of the transcription terminator sequence just after the transcription start site via a non-homologous end-joining mechanism.^181^ In contrast to the base complementary pairing-based nucleic acid techniques discussed above, small molecules can bind to or block lncRNAs, affect their spatial organization, or disrupt their interactions with proteins as secondary or tertiary structures within lncRNAs, which can serve as targets for stably binding to ligand molecules.^174^^,^^182^

Exosomes can carry a substantial amount of therapeutic cargo, which can be engineered to knock down or restore pathogenic lncRNA expression in lncRNA-dominated diseases. Those derived from immune cells, tumor cells, mesenchymal stem cells, and external sources are most commonly applied in drug delivery systems. The lncRNA DARS-AS1 is found to be a tumor promoter overexpressing in triple-negative breast cancer tissues. The application of exosomes from organisms as drug delivery vehicles to load DARS-AS1 siRNA inhibits the growth and metastasis of triple-negative breast cancer tumors.^183^ The RNA and protein in urinary exosomes are more stable than the soluble RNA and protein in urine, which have wide application prospects in the diagnosis of urologic diseases.^184^ When renal damage is present, the expression of miRNA-200c, miRNA-24, and miRNA-16 in urinary exosomes is increased, and their expansion is correlated with a reduction in target mRNA in the medulla of the glomerulus. TGF-β1 controls the production of miRNA-351 and miRNA-125a in exosomes after healing of the renal damage. Hence, the shift in miRNA in urinary exosomes that occurs during AKI presumably gives hints about the propensity for AKI to progress to CKD.^185^

In addition, other common lncRNA delivery systems include adenoviral vectors, liposomes, and nanoparticles. For instance, delivery of LINC00589 via mesoporous silica nanoparticles can inhibit peritoneal metastasis in gastric cancer.^186^ Plasmid-SLERCC@PDA@MUC12 nanoparticles encapsulating plasmid-encoding lncRNA-SLERCC effectively attenuate the growth of renal cell carcinoma metastases.^187^ Therefore, utilizing a combination of various delivery systems is a novel choice because each has its strengths and drawbacks.

## Discussion and outlook

The discovery of the mechanisms of action of exosomal and circulating lncRNAs on kidney damage is hastening the development of novel diagnostic and therapeutic tools. Exosomes are less immunogenic and more durable *in vivo* compared with viral and non-viral nanocarriers, which can pass through various biological barriers and carry a variety of therapeutic medicines. However, since some components (*e.g.*, microvesicles, chylomicrons, and high-density lipoproteins) are close to exosomes in size range, the exosomal isolation and purification procedures must be further optimized. Many lncRNA targeting strategies still have off-target effects that need to be eliminated. Due to the insufficient data from clinical trials, it remains unclear if lncRNA medications are effective and safe for use. However, exosomal lncRNAs still have great potential for application, with both opportunities and challenges. As mechanistic studies continue and new technologies are emerging, exosomal lncRNAs are anticipated to offer new alternatives for precision medicine soon.

## CRediT authorship contribution statement

**Minhui Zheng:** Writing - Original Draft, Conceptualization, Visualization. **Zixuan Yang:** Investigation, Writing - Review & Editing. **Lei Shi:** Investigation, Writing - Review & Editing. **Liyuan Zhao:** Investigation, Writing - Review & Editing. **Kelan Liu:** Investigation, Writing - Review & Editing. **Naping Tang:** Supervision, Conceptualization, Writing - Review & Editing.

## Conflict of interests

The authors declared no conflict of interests.
